# A simulation study to examine the impact of recombination on phylogenomic inferences under the multispecies coalescent model

**DOI:** 10.1111/mec.16433

**Published:** 2022-04-04

**Authors:** Tianqi Zhu, Tomáš Flouri, Ziheng Yang

**Affiliations:** ^1^ Institute of Applied Mathematics Academy of Mathematics and Systems Science Chinese Academy of Sciences Beijing China; ^2^ Key Laboratory of Random Complex Structures and Data Science Academy of Mathematics and Systems Science Chinese Academy of Sciences Beijing China; ^3^ 4919 Department of Genetics, Evolution and Environment University College London London UK

**Keywords:** BPP, introgression, MSci, multispecies coalescent, recombination, species delimitation, species tree

## Abstract

Phylogenomic analyses under the multispecies coalescent model assume no recombination within locus and free recombination among loci. Yet, in real data sets intralocus recombination causes different sites of the same locus to have different genealogical histories so that the model is misspecified. The impact of recombination on various coalescent‐based phylogenomic analyses has not been systematically examined. Here, we conduct a computer simulation to examine the impact of recombination on several Bayesian analyses of multilocus sequence data, including species tree estimation, species delimitation (by Bayesian selection of delimitation models) and estimation of evolutionary parameters such as species divergence and introgression times, population sizes for modern and extinct species, and cross‐species introgression probabilities. We found that recombination, at rates comparable to estimates from the human being, has little impact on coalescent‐based species tree estimation, species delimitation and estimation of population parameters. At rates 10 times higher than the human rate, recombination may affect parameter estimation, causing positive biases in introgression times and ancestral population sizes, although species divergence times and cross‐species introgression probabilities are estimated with little bias. Overall, the simulation suggests that phylogenomic inferences under the multispecies coalescent model are robust to realistic amounts of intralocus recombination.

## 1 INTRODUCTION

Advancements in sequencing technologies and accumulation of genomic sequence data have brought population genetics and phylogenetics into the genomics age. The availability of genomic data from multiple closely related species makes it possible to address many exciting biological questions, and the multispecies coalescent (MSC) model (Rannala & Yang, 2003) has emerged as the natural inference framework, as it accounts for both species divergences and the coalescent process in each species on the phylogeny. By treating the unobserved genealogical trees at the sampled loci as latent variables, the model makes use of information in the gene trees while naturally accommodating phylogenetic uncertainties. The MSC has been used to estimate population parameters such as species divergence times and effective population sizes (Burgess & Yang, 2008; Ogilvie et al., 2017; Rannala & Yang, 2003), to infer species phylogeny despite widespread genealogical fluctuations across the genome (Heled & Drummond, 2010; Liu & Pearl, 2007; Rannala & Yang, 2017; Yang & Rannala, 2014), and to identify and delimit species (Yang & Rannala, 2010, 2017). The MSC has also been extended to accommodate cross‐species gene flow, either continuous migration in the isolation‐with‐migration or IM models (Dalquen et al., 2017; Hey, 2010b; Hey et al., 2018; Zhu & Yang, 2012) or pulses of introgression/hybridization in the MSC‐with‐introgression or MSci models (Flouri et al., 2020; Wen & Nakhleh, 2018; Zhang et al., 2018). See Xu and Yang (2016), Kubatko (2019), Rannala et al. (2020), and Jiao et al. (2021) for recent reviews of the MSC and its applications.

The basic MSC model assumes independent genealogical trees at different loci and a common gene tree for all sites in the sequence at any locus. Here, the term locus refers to a short genomic segment, which may not be protein‐coding or even functional, although exonic data have been successfully used in such analysis (Shi & Yang, 2018; Thawornwattana et al., 2018, 2021). Such multilocus data sets have been widely used in traditional population genetic studies, and the loci may and may not correspond to protein‐coding genes (or silent sites in the coding genes, e.g. Takahata, 1986; Hudson et al., 1987; Ohta, 1995; Takahata et al., 1995). In analysis of modern genomic data, a common approach to generating such data is to sample short segments from the genome that are far apart (e.g. Beerli & Felsenstein, 2001; Burgess & Yang, 2008; Dalquen et al., 2017; Hey et al., 2018; Lohse et al., 2011; Nielsen & Wakeley, 2001; Wang & Hey, 2010). For example, each segment may be 100–2000 bps long and separated by at least 2 kb or 10 kb. The large gap means that different loci have approximately independent coalescent histories because of recombination between loci, while intralocus recombination is unlikely in a short segment. In analysis of data from different populations of the same species or from closely related species with low mutation rates, longer segments (of 2–10 kb, say) are sometimes used to ensure the presence of variable sites at each locus, with the four‐gamete test (Hudson & Kaplan, 1985) applied to filter out genomic regions likely affected by recombination (Hey, 2010a; Hey et al., 2018; Lohse & Frantz, 2014). Another approach to generating multilocus phylogenomic data sets is targeted sequence capture, also called reduced‐representation sequencing. This is an increasingly popular alternative to the more costly whole‐genome sequencing. Example data sets produced using this approach include RAD‐seq (Eaton & Ree, 2013; Rubin et al., 2012), ddRAD‐seq (Ali et al., 2016), exomes, transcriptomes, ultraconserved elements (UCEs; Faircloth et al., 2012), anchored hybrid enrichment (AHE; Lemmon et al., 2012), conserved nonexonic elements (CNEEs; Edwards et al., 2017) and rapidly evolving long exon capture (RELEC; Karin et al., 2020). The targeted genomic segments are typically 100–1000 bps long and are treated as independent loci in coalescent‐based phylogenomic analysis.

While both assumptions of no intralocus recombination and of free interlocus recombination are violated in real data sets, the former may be more of a concern. When the genealogical trees for different loci are correlated due to linkage, a model ignoring linkage may still fit the true gene trees at individual loci, and the impact of assuming independence should be an overstatement of the information content in the data or too narrow confidence intervals. As phylogenomic data sets often include thousands of loci and the confidence intervals are narrow anyway, this effect may not be very important. In contrast, intralocus recombination causes different sites of the same locus to have different histories, while the model ignoring recombination assumes that all sites share the same gene tree and branch lengths so that incorrect gene trees are applied to at least some sites in the alignment. In particular, recombination may create chimeric sequences that look very different from nonrecombinant sequences, leading to long branches in the gene tree and exaggerated levels of sequence divergence. Thus, the assumption of no intralocus recombination may be expected to be more damaging than that of free recombination among loci.

Few studies have examined the impact of intralocus recombination on coalescent‐based phylogenomic analyses, even though its importance has been hotly debated (see, e.g. Edwards et al., 2016; Gatesy & Springer, 2014). Wall (2003) incorporated recombination in a simulation‐based approximate inference method under the basic MSC model (Takahata et al., 1995; Yang, 2002) to estimate divergence times and ancestral population sizes among the human being and the great apes, noting that intralocus recombination causes overestimation of divergence times and underestimation of ancestral population sizes (Takahata & Satta, 2002). Zhu and Yang (2012) conducted a small simulation to examine the impact of recombination on a likelihood‐ratio test of gene flow using the maximum‐likelihood (ML) program 3s, which compares the null hypothesis of MSC with no gene flow against the alternative hypothesis of MSC with migration. The false‐positive rate was found to be low except when the recombination rate was orders of magnitude higher than estimated rates from the human being. Lanier and Knowles (2012) conducted a simulation study to examine the impact of recombination on species tree estimation using the heuristic method STEM (Kubatko et al., 2009) and the Bayesian method *beast (Heled & Drummond, 2010). Species tree estimation was found to be robust to recombination even at levels far higher than estimates from real data. Indeed, recombination was the least important factor affecting species tree estimation, and far less important than the number of sequences and the number of loci in the data set. However, the study had very limited scope, with at most nine sequences or nine loci, whereas modern phylogenomic studies routinely include thousands of loci. The study also used an unconventional experimental design, sampling species trees and parameter values at random for each simulation replicate. The impact of recombination is expected to depend on the species tree shape and species divergence times, and frequentist simulation with replicate data sets generated on a fixed species tree with fixed parameter values is preferable. We note that STEM uses estimated gene tree topologies and coalescent times (branch lengths) without accommodating their sampling errors, and is in particular sensitive to errors in gene tree branch lengths (Degnan, 2018; Leaché & Rannala, 2011), and similarly, the early version of *beast had very limited capability. Lastly, Lohse and Frantz (2014) used simulation to evaluate the impact of recombination on their inference of Neandertal admixture into Eurasian populations, and found that their comparison of models of admixture and ancestral structure was robust to realistic levels of recombination (around 1.3 cM/Mb).

The last decade has seen considerable computational improvements and algorithmic breakthroughs in Bayesian implementations of the MSC model, either with or without accommodating gene flow, making it possible to analyse large genomic data sets with over 10,000 loci (Shi & Yang, 2018; Thawornwattana et al., 2018, 2021). A range of inference problems have been addressed, including species tree estimation despite conflicting gene trees (Rannala & Yang, 2017; Yang & Rannala, 2014), species delimitation (Yang & Rannala, 2010, 2014) and estimation of population parameters such as species divergence times, population sizes for extant and extinct species, and the rates and times of ancient introgression events (Burgess & Yang, 2008; Flouri et al., 2020). There is thus a need to evaluate the impact of recombination on inference using modern software programs and realistically sized data sets. In this paper, we conduct a simulation study to examine the impact of recombination on several Bayesian inference problems under the MSC model using multilocus phylogenomic data. We use estimates of recombination rates from the human being as a guide to simulate data sets with recombination and analysed them assuming no recombination to examine the robustness of the analysis. We conducted three sets of simulations to examine three inference problems: (i) estimation of the species tree topology under the MSC model with no gene flow, (ii) delimitation of species boundaries through Bayesian model selection, and (iii) estimation of population parameters under the MSC model with introgression (MSci), including species divergence times, population sizes and cross‐species introgression probabilities. The Bayesian program bpp was used, which is a full‐likelihood implementation of the MSC model with and without gene flow applied to multilocus genomic data sets (Flouri et al., 2018). From a statistical point of view, the method is expected to have optimal properties, compared with heuristic methods based on data summaries such as the estimated gene tree topologies. The simulation results should serve as a useful guide for empirical studies in which real genomic data sets are analysed with recombination ignored.

## 2 MATERIALS AND METHODS

### 2.1 A01 species tree estimation

**FIGURE 1:**
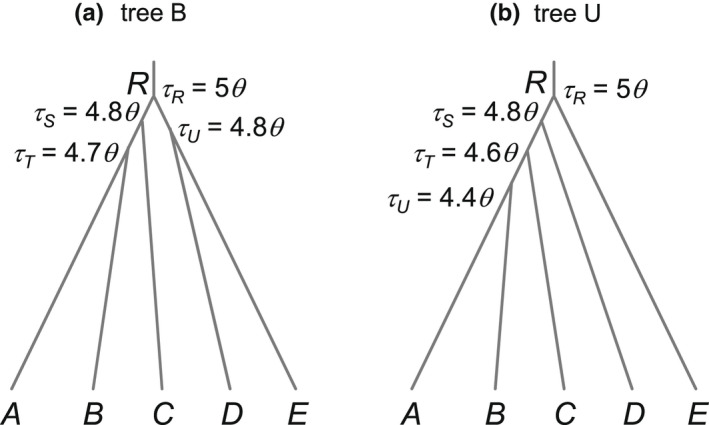
(a) Balanced species tree B and (b) unbalanced species tree U for five species, used to simulate data for species tree estimation under the MSC model. Both species divergence times (*τs*) and population sizes (*θs*) are measured by the expected number of mutations per site. Two values are used for θ: 0.0025 and 0.01, to mimic low and high mutation rates

In the first set of simulations, we examined the impact of recombination on the estimation of species tree topology under the MSC model. Data were simulated by using the program MS (Hudson, 2002) to generate the genealogical trees with branch lengths (coalescent times) for different sequence segments at each locus and then using seq‐gen (Rambaut & Grassly, 1997) to generate sequence alignments under the JC mutation model (Jukes & Cantor, 1969). Sequences at the tips of the gene tree constituted the data at the locus. We assumed two challenging species trees, with short internal branches (Figure 1). In the balanced species tree B, the divergence times were $\tau_{R}=5\theta$, $\tau_{S}=4.8\theta$, $\tau_{T}=4.7\theta$ and $\tau_{U}=4.8\theta$. In the unbalanced species tree U, the parameters were $\tau_{R}=5\theta$, $\tau_{S}=4.8\theta$, $\tau_{T}=4.6\theta$ and $\tau_{U}=4.4\theta$. Here, the population size parameter is defined as $\theta =4N_{e}\mu$, with $N_{e}$ to be the effective population size and μ the mutation rate per site per generation. This is also known as heterozygosity and varies hugely among species, with previous estimates for extant animal and plant species covering a broad range (0.0005–0.02; Zhang & Hewitt, 2003). We used two values for θ: 0.0025 and 0.01, to represent different species. In our experiment, species divergence times (*τs*) are proportional to θ so that the different *θs* may also mimic different types of genomic regions with different mutation rates (e.g. ultraconserved elements or UCEs versus introns).

We chose recombination rates based on estimates for the human being, at r=0.37 cM/Mb (Arnheim et al., 2007) or 1.13 cM/Mb from segregation analysis on pedigrees (Kong et al., 2002). With the effective population size of $N_{e}=10^{4}$, those estimates translate to the population recombination rates of $\rho =4N_{e}r=0.148$ and 0.452 per generation per kb. We used three values: ρ=0.05,0.5, and 5 per generation per kb, with the intermediate value to be slightly higher than the estimates from the human being, while the other two are 10 times smaller or larger. We note that recombination rate varies among species (see Discussion). The average rate for mice is at 0.5 cM/Mb (Kauppi et al., 2004), while in *Drosophila*, estimated rates for autosomes are mostly 0–1 cM/Mb, with 2.3 cM/Mb considered to be high rates (Singh et al., 2005).

We sampled either S=2 or 8 sequences per species at each locus, with the sequence length to be 500 sites. Each replicate data set consisted of L=40 or 160 loci. The number of replicates was 100. The total number of simulated data sets, over all combinations of the species tree, the number of sequences per species (S), the number of loci (L), the mutation rate (θ) and the recombination rate (ρ), was 2×2×2×2×3×100=4800.

Each replicate data set was analysed using bpp version 4 to estimate the species tree (Flouri et al., 2018; Rannala & Yang, 2017). This is the A01 analysis of Yang (2015). The correct mutation model (JC) was assumed, but intralocus recombination was ignored. Inverse‐gamma priors were assigned on the age of the root on the species tree ($\tau_{0}=\tau_{R}$) and the population size parameters (θ): $\tau_{0}\;∼$ IG(3, 0.025) and θ ~ IG(3, 0.005) for θ=0.0025; and $\tau_{0}$ ~ IG(3, 0.1) and θ ~ IG(3, 0.02) for θ=0.01. Here, the inverse‐gamma prior IG(α,β) has the mean β/(α‐1), while the shape parameter α=3 means that the prior is diffuse. Independent θ parameters were assigned to different branches on the species tree, but they were integrated out analytically through the conjugate inverse‐gamma priors to help with MCMC mixing (Flouri et al., 2018). Pilot runs were used to determine the suitable settings for the MCMC, with convergence assessed by running the same analysis multiple times and confirming consistency between runs (Flouri et al., 2018; Yang, 2015). Then, the same setting was used to analyse all replicates. We used 32,000 iterations for burn‐in, after which we took $10^{5}$ samples, sampling every 5 iterations. Analysis of each data set took ~20 min on a single core for small data sets of 40 loci and 10 sequences per locus or ~20 h for large data sets of 160 loci and 40 sequences per locus, with longer running time for more divergent data simulated using the higher mutation rate (θ=0.01).

### 2.2 A11 species delimitation

**FIGURE 2:**
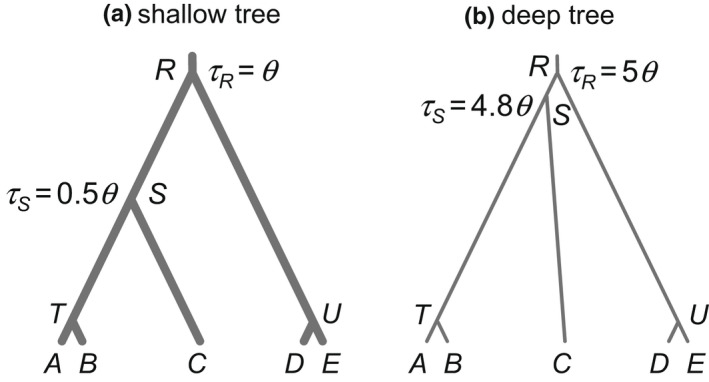
Species trees or MSC models used in the simulation for species delimitation, with five populations (A,B,C,D and E) and three distinct species (AB, C and DE) in the true model. Data are simulated by assuming the tree of five populations with $\tau_{T}$ and $\tau_{U}$ set to very small values ($=10^{‐50}\theta$), and then analysed to infer both the species delimitation and species phylogeny (the A11 analysis; Yang, 2015). In (a), the shallow tree, $\tau_{R}=\theta$ and $\tau_{S}=0.5\theta$, while in (b), the deep tree, $\tau_{R}=5\theta$ and $\tau_{S}=4.8\theta$. The thickness of the branches indicates the population sizes (*θs*) relative to the species divergence times (*τs*). Two values are used for θ: 0.0025 and 0.01

In the second set of simulation, we examined the impact of recombination on species delimitation under the MSC model (Yang & Rannala, 2010, 2014). We used two models/trees, referred to as the shallow tree and the deep tree, respectively, each with three species (AB, C and DE) and five populations (A,B,C,Dand E; Figure 2). In the shallow tree, $\tau_{R}=\theta$, $\tau_{S}=0.5\theta$, while in the deep tree, $\tau_{R}=5\theta$and $\tau_{S}=4.8\theta$. Sequence data from five populations were simulated using the species trees of Figure 2, with $\tau_{T}$ and $\tau_{U}$ fixed at ≈0. At each locus, S=2or 8 sequences are sampled from each population. Other parameter settings were as before. With 100 replicates, the total number of data sets simulated was 4800.

The data sets were analysed to infer the species delimitation and species phylogeny. This is the A11 analysis of Yang (2015). We did not assess the impact of misassignment of individual sequences to populations. Instead, the correct assignment was assumed, with bpp comparing different models of merging the five populations into species and rearranging the phylogenetic relationships if three or more species were inferred (Yang & Rannala, 2014). Similarly, we integrated out *θs* analytically to improve mixing. The bpp program summarizes the MCMC sample to produce posterior probabilities for different models and for different delimitations. The maximum *a posteriori* probability (MAP) model constitutes the best inferred model from the data set. We also used the posterior probabilities for the true model and true delimitation to assess performance. Running time for analysing one replicate data set was ~20 min for small data sets of 40 loci and 10 sequences per locus or 12 h for large data sets of 160 loci and 40 sequences per locus.

### 2.3 A00 Estimation of population parameters under the MSci model

**FIGURE 3:**
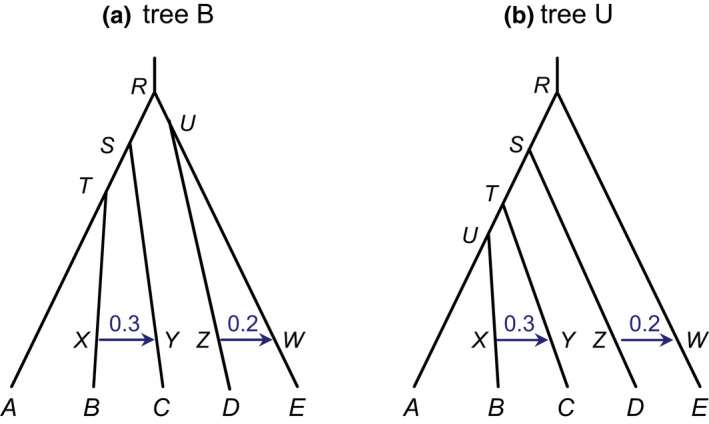
Two introgression (MSci) models used in the simulation to examine Bayesian parameter estimation. The parameters for tree B are $\tau_{R}=5\theta$, $\tau_{S}=4\theta$, $\tau_{T}=3\theta$, $\tau_{U}=4.5\theta$, $\tau_{X}=\tau_{Y}=\theta$ and $\tau_{Z}=\tau_{W}=\theta$, while those for tree U are $\tau_{R}=5\theta$, $\tau_{S}=4\theta$, $\tau_{T}=3\theta$, $\tau_{U}=2.5\theta$, $\tau_{X}=\tau_{Y}=\theta$ and $\tau_{Z}=\tau_{W}=\theta$. In both trees, the introgression probabilities are $\varphi_{Y}=0.3$ and $\varphi_{W}=0.2$. Two values are used for θ: 0.0025 and 0.01

In the third set of simulation, we evaluated the impact of intralocus recombination on Bayesian estimation of population parameters under the MSci model, including species divergence times (τs), (effective) population sizes (*θs*) and introgression probabilities at hybridization/introgression nodes. We simulated data under the two MSci models of Figure 3, referred to as trees B and U, each involving two introgression events, with introgression probabilities $\varphi_{Y}=0.3$ and $\varphi_{W}=0.2$ (Flouri et al., 2020). The introgression probability in the MSci model specifies the contributions of the two parental populations to each hybridizing species at the time of introgression; for example, in tree B (Figure 3a), species Y (ancestral to C) was an admixture population with $\varphi_{Y}=30\%$ contribution from the parental species TX and $1‐\varphi_{Y}=70\%$ from the other parent SY. The divergence times are given in the figure legend. Again, we simulated gene trees and sequence alignments using bpp under the JC model. A total of 4800 replicate data sets were generated. Each data set was then analysed to estimate the parameters under the correct model. Priors on τs and θs are as before, while φs are assigned the uniform prior U(0,1).

The posterior mean for each parameter provides the point estimate, while the 95% highest probability density (HPD) credibility intervals (CIs) measure the uncertainty. We calculated the bias in parameter estimate, the 95% HPD CI width and the relative root mean square error (rRMSE), defined as

(1) $$\text{rRMSE}=\frac{1}{\phi }{\left[\frac{1}{R}\sum_{i=1}^{R}{\left({\hat{\phi }}_{i}‐\phi \right)}^{2}\right]}^{\frac{1}{2}},$$

where ϕ is the true value of any parameter, and ${\hat{\phi }}_{i,}$ its estimate in the ith replicate data set, with i=1,⋯,R=100. For example, rRMSE = 0.1 means that the mean square error is 10% of the true value. The rRMSE reflects both the bias and the variance in the estimate.

Running time for analysing one replicate data set was ~25 min for small data sets of 40 loci and 10 sequences per locus or ~12 h for large data sets of 160 loci and 40 sequences per locus.

## 3 RESULTS

### 3.1 A01 species tree estimation

**FIGURE 4:**
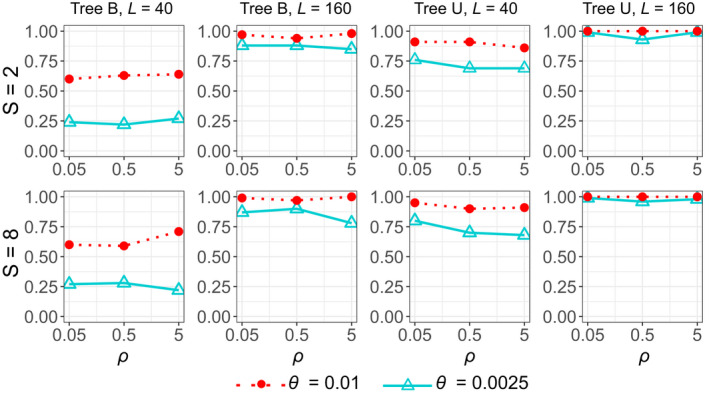
(A01) Probability of recovering the true species tree of Figure 1 plotted against the recombination rate (ρ)

Replicate data sets were simulated using species trees B and U of Figure 1 and analysed using bpp to infer the species tree topology. The A01 analysis (Yang, 2015) produces a posterior distribution of species trees. The MAP species tree may be considered the best point estimate of the true species tree (Yang, 1996). The probability that the MAP tree is the true tree (i.e. that the true species tree is recovered) measures the efficiency of the method (Figure 4). This either increased or decreased when the recombination rate changed by two orders of magnitude, but the differences were small, no larger than random sampling errors expected from our use of 100 replicates. For example, the probability that the MAP tree matches the true species was 0.24, 0.22 and 0.27 for ρ=0.05,0.5 and 5, respectively, in the least informative data sets simulated using species tree B with S=2, L=40, at the low mutation rate θ=0.0025. Similarly, the probabilities of recovering the true clades R (the whole species tree), S, T and U on the true species tree (Figure 1a and b) varied very little among the three recombination rates used (Table 1), and the average posterior probabilities for the true clades (Table 2) were very similar for the three recombination rates as well. Overall, recombination, at the rates considered here, had little impact on species tree estimation. The average number of recombination events that occurred at each locus should be proportional to the recombination rate ρ, and was 0.7–0.9 at the low rate, 7–9 at the medium rate and 66–81 at the high rate (Table 3). While at the low rate about a half of the loci had no recombination events, this proportion dropped to 0% at the medium or high rates.

**TABLE 1:** (A01) Proportions of simulated replicates in which the true subtrees R, S, T and U in species trees B and U of Figure 1 are recovered in the MAP species tree

S	L	Low rate (θ=0.0025)			High rate (θ=0.01)		
		ρ=0.05	ρ=0.5	ρ=5	ρ=0.05	ρ=0.5	ρ=5
Tree B							
2	40	0.24, 0.52, 0.56, 0.63	0.22, 0.47, 0.58, 0.57	0.27, 0.48, 0.63, 0.67	0.60, 0.72, 0.76, 0.87	0.63, 0.72, 0.75, 0.87	0.64, 0.72, 0.76, 0.86
	160	0.88, 0.94, 0.94, 0.93	0.88, 0.92, 0.93, 0.96	0.85, 0.88, 0.90, 0.96	0.97, 0.97, 0.97, 1.00	0.94, 0.94, 0.94, 1.00	0.98, 0.98, 0.98, 1.00
8	40	0.27, 0.46, 0.56, 0.71	0.28, 0.54, 0.63, 0.65	0.22, 0.52, 0.60, 0.62	0.60, 0.75, 0.79, 0.80	0.59, 0.70, 0.72, 0.88	0.71, 0.80, 0.83, 0.89
	160	0.87, 0.90, 0.90, 0.96	0.90, 0.92, 0.93, 0.98	0.78, 0.92, 0.95, 0.86	0.99, 0.99, 0.99, 1.00	0.97, 0.97, 0.97, 1.00	1.00, 1.00, 1.00, 1.00
Tree U							
2	40	0.76, 0.76, 0.89, 0.92	0.69, 0.69, 0.91, 0.93	0.69, 0.69, 0.86, 0.94	0.91, 0.91, 0.95, 0.97	0.91, 0.91, 0.96, 0.97	0.86, 0.86, 1.00, 1.00
	160	0.99, 0.99, 1.00, 1.00	0.93, 0.93, 1.00, 1.00	0.99, 0.99, 0.99, 1.00	1.00, 1.00, 1.00, 1.00	1.00, 1.00, 1.00, 1.00	1.00, 1.00, 1.00, 1.00
8	40	0.80, 0.80, 0.88, 0.93	0.70, 0.70, 0.85, 0.89	0.68, 0.68, 0.80, 0.88	0.95, 0.95, 0.96, 0.98	0.90, 0.90, 0.96, 0.96	0.91, 0.91, 0.97, 0.98
	160	0.99, 0.99, 1.00, 1.00	0.96, 0.96, 1.00, 1.00	0.98, 0.98, 1.00, 1.00	1.00, 1.00, 1.00, 1.00	1.00, 1.00, 1.00, 1.00	1.00, 1.00, 1.00, 1.00

**TABLE 2:** (A01) Average posterior probabilities for true subtrees R (the whole tree), S, T, and U in species trees B and U of Figure 1

S	L	Low rate (θ=0.0025)			High rate (θ=0.01)		
		ρ=0.05	ρ=0.5	ρ=5	ρ=0.05	ρ=0.5	ρ=5
Tree B							
2	40	0.16, 0.37, 0.48, 0.63	0.18, 0.43, 0.53, 0.55	0.18, 0.37, 0.51, 0.64	0.42, 0.55, 0.64, 0.81	0.45, 0.55, 0.63, 0.82	0.47, 0.59, 0.69, 0.81
	160	0.71, 0.82, 0.85, 0.87	0.70, 0.79, 0.84, 0.89	0.66, 0.74, 0.81, 0.90	0.90, 0.91, 0.91, 1.00	0.89, 0.90, 0.90, 1.00	0.95, 0.95, 0.95, 1.00
8	40	0.17, 0.35, 0.46, 0.66	0.18, 0.40, 0.54, 0.64	0.17, 0.40, 0.53, 0.63	0.47, 0.63, 0.70, 0.75	0.44, 0.59, 0.67, 0.80	0.49, 0.64, 0.70, 0.81
	160	0.68, 0.78, 0.79, 0.88	0.70, 0.79, 0.81, 0.90	0.64, 0.82, 0.85, 0.80	0.92, 0.93, 0.93, 0.99	0.91, 0.92, 0.92, 0.99	0.96, 0.96, 0.96, 1.00
Tree U							
2	40	0.51, 0.51, 0.70, 0.83	0.48, 0.48, 0.70, 0.82	0.48, 0.48, 0.69, 0.83	0.74, 0.74, 0.84, 0.90	0.76, 0.76, 0.88, 0.93	0.72, 0.72, 0.91, 0.95
	160	0.92, 0.92, 0.99, 0.99	0.89, 0.89, 0.99, 1.00	0.95, 0.95, 0.99, 1.00	1.00, 1.00, 1.00, 1.00	1.00, 1.00, 1.00, 1.00	0.99, 0.99, 1.00, 1.00
8	40	0.53, 0.53, 0.72, 0.83	0.48, 0.48, 0.67, 0.79	0.50, 0.50, 0.69, 0.81	0.81, 0.81, 0.88, 0.93	0.74, 0.74, 0.85, 0.90	0.75, 0.75, 0.89, 0.94
	160	0.94, 0.94, 0.99, 0.99	0.93, 0.93, 1.00, 1.00	0.92, 0.92, 0.99, 1.00	1.00, 1.00, 1.00, 1.00	1.00, 1.00, 1.00, 1.00	0.99, 0.99, 1.00, 1.00

**TABLE 3:** Average number of recombination events per locus and the average proportion of loci with no recombination (in parentheses)

Tree	ρ	S=2		S=8	
		θ=0.0025	θ=0.01	θ=0.0025	θ=0.01
A01 analysis					
Tree B (Figure 1a)	0.05	0.7 (0.49)	0.7 (0.48)	0.9 (0.42)	0.9 (0.41)
	0.5	7.2 (0.00)	7.2 (0.00)	8.7 (0.00)	8.7 (0.00)
	5	67.6 (0.00)	67.8 (0.00)	80.5 (0.00)	80.6 (0.00)
Tree U (Figure 1b)	0.05	0.7 (0.49)	0.7 (0.49)	0.9 (0.43)	0.9 (0.42)
	0.5	7.1 (0.00)	7.1 (0.00)	8.5 (0.00)	8.6 (0.00)
	5	66.5 (0.00)	66.4 (0.00)	79.3 (0.00)	79.5 (0.00)
A11 analysis					
Shallow (Figure 2a)	0.05	0.5 (0.63)	0.5 (0.63)	0.6 (0.57)	0.6 (0.57)
	0.5	4.7 (0.01)	4.7 (0.01)	5.7 (0.00)	5.7 (0.00)
	5	45.2 (0.00)	45.1 (0.00)	53.9 (0.00)	53.6 (0.00)
Deep (Figure 2b)	0.05	0.2 (0.85)	0.2 (0.85)	0.3 (0.78)	0.3 (0.78)
	0.5	1.6 (0.00)	1.6 (0.00)	2.5 (0.09)	2.5 (0.09)
	5	15.8 (0.00)	15.9 (0.00)	24.6 (0.00)	24.5 (0.00)
A00 analysis					
Tree B (Figure 3a)	0.05	0.6 (0.54)	0.6 (0.54)	0.8 (0.46)	0.8 (0.46)
	0.5	6.2 (0.00)	6.2 (0.00)	7.8 (0.00)	7.7 (0.00)
	5	58.8 (0.00)	58.8 (0.00)	72.4 (0.00)	72.4 (0.00)
Tree U (Figure 3b)	0.05	0.6 (0.57)	0.6 (0.56)	0.7 (0.49)	0.7 (0.48)
	0.5	5.7 (0.00)	5.7 (0.00)	7.2 (0.00)	7.3 (0.00)
	5	54.3 (0.00)	54.1 (0.00)	67.9 (0.00)	67.9 (0.00)

Note

The results are averages over the 100 replicate data sets of L = 160 loci. The expected number of recombinations increases with the number of sequences (S) and is proportional to the recombination rate (ρ), but independent of the mutation rate (θ).

We also note that trees B and U of Figure 1 are challenging species trees because of the extremely short internal branches. The average coalescent time between two sequences sampled from the same species with population size θ is $\frac{1}{2}\theta$. In comparison, the time gaps between speciation events in the species trees of Figure 1 (0.2θ or 0.1θ) are much shorter, representing a scenario of very rapid successions of speciation events, resulting in species trees that are hard to resolve. For easy trees with long internal branches, recombination is expected to be even less important.

The other factors considered in the simulation (the number of sequences S sampled per locus per species, the mutation rate θ and the number of loci L) had far more impact on species tree estimation than recombination (Table 4). Performance improved slightly when the number of sequences increased from S=2 to 8 (Figure 4, Tables 1 and 2). Increasing the mutation rate from θ=0.0025 to 0.01 improved performance dramatically, while the greatest improvement came from the increase in the number of loci (from L=40 to 160). For example, the probability of recovering the balanced species tree B at the low recombination rate (ρ=0.05) was 0.24 at S=2, θ=0.0025 and L=40 (Table 1). This increased to 0.27 with the number of sequences increased by fourfold (S=8), to 0.60 when the mutation rate increased by fourfold (θ=0.01), and to 0.88 when the number of loci increased by fourfold (L=160). The patterns were similar when the unbalanced species tree U (Figure 1) was used in the simulation (Figure 4, Tables 1 and 2). Those results are consistent with the previous simulation study of Huang et al. (2020), which examined the relative importance of various factors that influence the information content in the data on species tree estimation.

**TABLE 4:** Relative importance of different factors in different inference problems

Analysis	Influence
A01 (species tree under MSC)	L≻θ≻S≻ρ
A11 (species tree and species delimitation)	
Species tree	L≍θ≍S≻ρ
Delimitation	L≍S≻θ≻ρ
A00 (parameter estimation)	
θs for modern species	L≍S≻θ≻ρ
θs for ancestral species	L≻θ≻S≻ρ
τs	L≻θ≻S≻ρ
φs	L≻θ≍S≻ρ

Note

The factors are the number of loci (L), the number of sequences per species per locus (S), the mutation rate (θ) and the recombination rate (ρ). L≻S means the number of loci (L) has more impact on the information content in the data or on method performance than the number of sequences (S), while ≍ means that the two factors have similar effects.

We note that the posterior probability for the true species tree was often much lower than the probability that the true species tree was recovered. For example, at S=8, θ=0.01 and L=160, species tree B was recovered in 87% of the replicate data sets (Table 1), but the average posterior probability for the true tree was only 0.68 (Table 2). Note that in our simulation, the species tree and model parameters were fixed when the replicate data sets were generated, so we are evaluating the Frequentist properties of a Bayesian method. The results suggest that Bayesian posterior probabilities for species trees are conservatively judged by the Frequentist criterion.

### 3.2 A11 species delimitation

**FIGURE 5:**
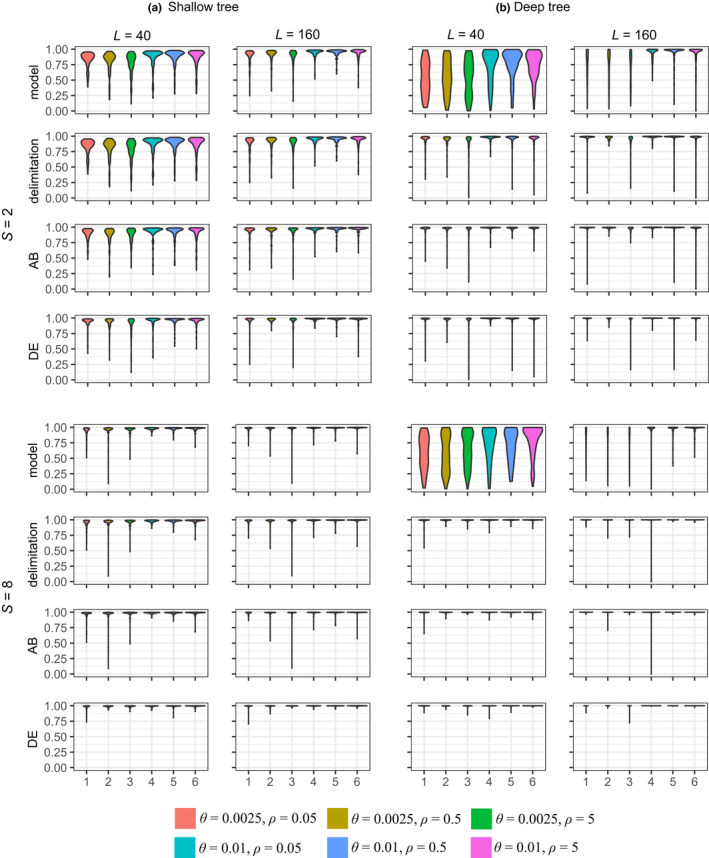
(A11) Violin plot of the posterior probabilities among the 100 replicates for the correct model (both delimitation and phylogeny), correct delimitation and correct delimited species AB and DE in the A11 analysis (joint species delimitation and species tree estimation, Yang, 2015). Delimitation C has posterior probability 1 in all data sets and is not shown. In (a), the shallow tree (Figure 2), $\tau_{R}=\theta$ and $\tau_{S}=0.5\theta$, while in (b), the deep tree, $\tau_{R}=5\theta$ and $\tau_{S}=4.8\theta$. The number of sequences per locus per species is S=2 or 8. In each panel, there are six combinations of θ and ρ, as indicated in the key

We simulated data sets using the shallow and deep species trees of Figure 2 and run bpp to calculate the posterior probabilities for different species delimitation models, which are different instances of the MSC model and correspond to different ways of merging the five populations into distinct species, with the number of inferred species ranging from 1 to 5. This is the A11 analysis of Yang (2015). We considered the correct model to be recovered if the correct number of species (3), the correct delimited species (AB, C and DE) and the correct species phylogeny ((AB,C),DE) were all recovered. The probability that the MAP model matches the true model (with correct delimitation and correct phylogeny) was very similar for the three recombination rates, for every parameter setting (Table 5). For example, this was 0.98, 0.95 and 0.94 for ρ=0.05,0.5 and 5, respectively, for data simulated using the shallow tree with S=2 sequences per species, low mutation rate θ=0.0025 and L=40 loci, while the corresponding values for the deep tree were 0.68, 0.70 and 0.68 (Table 5). Similarly, the posterior probabilities for the true model (both the delimitation and the phylogeny), the true delimitation and the true species (AB and DE) were very similar among the three recombination rates (Figure 5). Overall recombination had minimal impact on species delimitation at the rates considered here. The average number of recombination events per locus was about 0.5, 5 and 50 for the three recombination rates, respectively, for the shallow tree, and was in the order of 0.2, 2 and 20 at the three rates for the deep tree (Table 3). At the medium or high rates, almost every locus had at least one recombination event.

**TABLE 5:** A11 probability of recovering the true model and true delimitation in the species delimitation simulation under models of Figure 2

S	L	Low rate (θ=0.0025)			High rate (θ=0.01)		
		ρ=0.05	ρ=0.5	ρ=5	ρ=0.05	ρ=0.5	ρ=5
Shallow tree							
2	40	0.98, 0.98	0.95, 0.95	0.94, 0.94	0.94, 0.94	0.97, 0.97	0.96, 0.96
	160	0.97, 0.97	0.99, 0.99	0.95, 0.95	1.00, 1.00	1.00, 1.00	0.98, 0.98
8	40	1.00, 1.00	0.98, 0.98	0.99, 0.99	1.00, 1.00	1.00, 1.00	1.00, 1.00
	160	1.00, 1.00	1.00, 1.00	0.99, 0.99	1.00, 1.00	1.00, 1.00	1.00, 1.00
Deep tree							
2	40	0.68, 0.97	0.70, 0.99	0.68, 0.96	0.85, 1.00	0.88, 0.99	0.91, 0.99
	160	0.91, 0.98	0.91, 1.00	0.85, 0.97	1.00, 1.00	0.97, 0.98	0.99, 0.99
8	40	0.69, 1.00	0.68, 1.00	0.73, 1.00	0.84, 1.00	0.85, 1.00	0.88, 1.00
	160	0.95, 1.00	0.87, 1.00	0.88, 1.00	0.99, 0.99	0.98, 1.00	1.00, 1.00

In contrast, the other factors considered in the simulation had far more impact than recombination (Table 4). The posterior probability for the true model or true delimitation increased with the increase in the number of sequences per species (from S=2 to 8), with the increase in mutation rate (from θ=0.0025 to 0.01) and with the increase in the number of loci (from L=40 to 160). It is noteworthy that the number of sequences per species was even more important than the mutation rate, although the number of loci was the most important factor (Table 4). The importance of the number of sampled sequences to species delimitation was noted before by Zhang et al. (2011).

The shallow and deep trees showed different patterns. In the shallow tree, species divergence times were comparable to average coalescent times $\left(\frac{1}{2}\theta \right)$ and it was challenging to delineate species boundaries. As a result, the posterior probabilities for the correct model and for the correct delimitation were nearly the same (Figure 5): as long as the correct species delimitation was recovered; the phylogeny and thus the whole model were reconstructed correctly as well. In the deep tree, species divergences were much older than the coalescent times, but the species arose in a quick succession of speciation events with short internal branches. As a result, it was easy to delimit species but hard to infer the phylogeny. Thus, in many small data sets with L=40 loci, the posterior probability for the correct delimitation was ~100%, but the posterior for the whole model was very low (Figure 5).

### 3.3 A00 Estimation of population parameters

**FIGURE 6:**
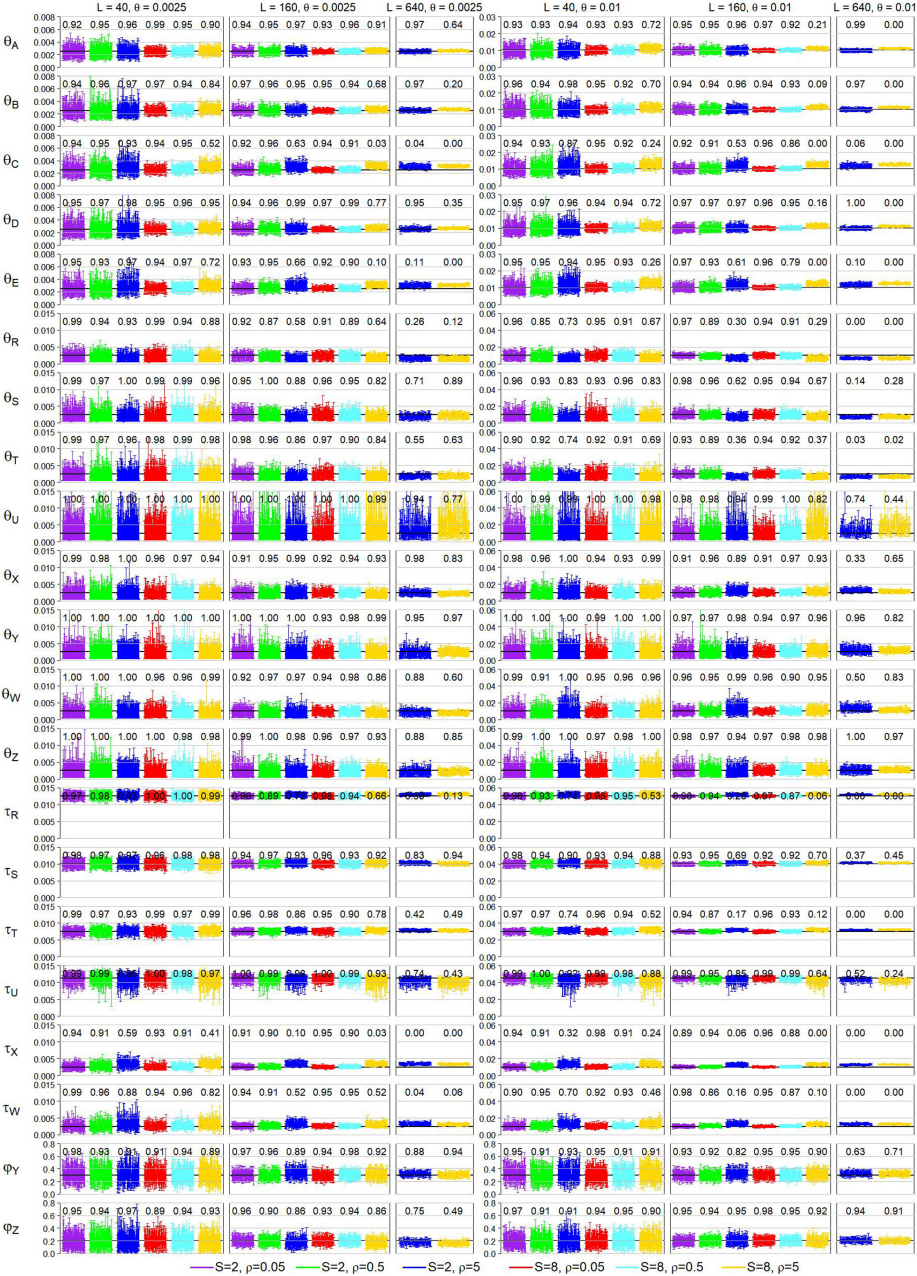
The Posterior 95% CIs and coverage for parameters under the MSci model for species tree B of Figure 3. Simulation for L=640 loci was done for the high recombination rate (ρ=5) only

We simulated data sets under the MSci model using the balanced species tree B and the unbalanced species tree U of Figure 3, involving two introgression events with introgression probabilities $\varphi_{Y}=0.3$ and $\varphi_{W}=0.2$. The data were then analysed using bpp with the MSci model fixed to estimate the 21 parameters in the model (6 τs, 13 θs, and 2 φs). The posterior HPD CIs among the 100 replicate data sets for each parameter setting are plotted in Figure 6 and Figure S1 for species trees B and U, respectively. The coverage probability for the CI is the proportion of replicate data sets in which the CI includes the true parameter value. The bias and relative root mean square error (rRMSE) in parameter estimates (posterior means) are shown in Tables S1 and S2.

Because the model is misspecified, parameter estimates are expected to be statistically inconsistent and to converge to incorrect parameter values when the amount of data increases. The limit of the maximum‐likelihood estimates (MLE, $\theta_{*}$) when the number of loci L approaches ∞, also known as the *best*‐*fitting* or *pseudo*‐*true parameter values*, minimizes the Kullback–Leibler divergence from misspecified model to the true model,

(2) $$D\left(\theta \right)=\int g\left(X;\theta_{0}\right)\log\frac{g(X;\theta_{0})}{f(X;\theta )}\;dX,$$

where X is the data (alignment at any locus), $g(X;\theta_{0})$ is the true density with the true parameter values $\theta_{0}$ under the correct model allowing for intralocus recombination, and f(X;θ) is the density under the misspecified model assuming no intralocus recombination. We expect that $\theta_{*}\neq \theta_{0}$. As a consequence when the data size increases, the CIs will become increasingly narrow, and the CI coverage will approach 0. The relevant question is then whether the ‘bias’ caused by recombination (or the difference between $\theta^{*}$ and $\theta_{0}$) is large enough to be of biological significance. Small biases may be tolerable even if the CI does not include the true value. We expect the information content in the data set to be mostly determined by the number of sequences (S), the mutation rate (θ) that affects the number of variable or informative sites in the alignment at each locus, and the number of loci (L), but should be similar at different recombination rates. Thus, the variance in parameter estimates is expected to be similar for different values of ρ (see discussion below for the case of very large ρ). Thus, we focus on the bias and CI coverage here.

For instance, at the high recombination rate (ρ=5), in the case of species tree B, high mutation rate (θ=0.01), S=8 sequences per species and L=160 loci, the average estimate of $\theta_{A}$ is 0.0111 (Table S1), with the CI coverage to be 21%, much lower than the nominal 95% (Figure 6). In replicate data set 1, the posterior mean was 0.0108 (with CI 0.0100–0.0116), whereas the true value was 0.01. Such a difference from the true value may be considered unimportant. Parameter $\theta_{C}$ was more poorly estimated than $\theta_{A}$, with the average to be 0.00127 (Table S1), and with the CI coverage to be 0%. In replicate 1, the posterior mean was 0.00119 (with CI 0.0110–0.0128). This was a slightly larger deviation from the true value, but the 20% relative bias may be tolerable given that population size varies hugely even between sister species. Note that both parameter estimates are inconsistent and the CI coverage will approach 0 when the number of loci approaches ∞. We thus emphasize the CI coverage for small‐ and medium‐sized data sets, and the bias $\theta_{*}‐\theta_{0}$ for large data sets in which the CI is very narrow.

Performance was extremely similar at the low and medium recombination rates (ρ=0.05 and 0.5) for all settings, with the bias to be within 10% of the true values (Figure 6 and Figure S1, Tables S1 and S2). This applies to all parameters (except $\theta_{U}$ on tree B), including population sizes for modern species ($\theta_{A}$–$\theta_{E}$), species divergence times ($\tau_{R}$–$\tau_{U}$) and introgression probabilities. Population sizes for ancestral species were hard to estimate and involve large CIs; in particular, $\theta_{U}$ on tree B was poorly estimated because the population corresponds to a short branch on the species tree (Table S1).

However, excessive recombinations (at the rate of ρ=5, ten times the human rate) caused more substantial biases in parameter estimates. Species divergence times ($\tau_{R}$, $\tau_{S}$, $\tau_{T}$ and $\tau_{U}$) were the least affected, with mostly a positive bias at ≤4% of the true value when ρ=0.5 (the human rate) or ≤7% of the true value when ρ=5 (10× the human rate) (Table S1). Population sizes for modern species ($\theta_{A}$, $\theta_{B}$ and $\theta_{D}$) were affected slightly more, with positive biases of up to 15% of the true values. Parameter $\theta_{C}$ was affected more than $\theta_{B}$ (and $\theta_{E}$ more than $\theta_{D}$), with positive biases of 20–30% when ρ=5, presumably because C and E were recipient populations for immigrants so that estimates of their sizes were easily affected by the time and strength of introgression. Introgression times ($\tau_{X}=\tau_{Y}$ and $\tau_{Z}=\tau_{W}$) had positive biases of 20–40% of the true value at ρ=5. Overestimation of $\tau_{X}=\tau_{Y}$ (or similarly of $\tau_{Z}=\tau_{W}$) was compensated by an overestimation of $\theta_{C}$ (or similarly of $\theta_{E}$). It is somewhat surprising that the introgression time was affected by recombination, but the introgression rate ($\varphi_{Y}$ or $\varphi_{W}$) was not (Table S1). Finally, excessive recombinations (at ρ=5) also caused underestimation of ancestral θs for the speciation nodes ($\theta_{R}$, $\theta_{S}$, and $\theta_{T}$), by about 20–30% (Table S1). The results are consistent with Wall (2003) and Lohse and Frantz (2014), who found that intralocus recombination caused underestimation of ancestral population sizes (θs) and overestimation of species divergence times (Takahata & Satta, 2002).

The average number of recombination events per locus was 0.6–0.8 for the low recombination rate, 6–8 for the medium rate and 60–80 for the high rate (Table 3). At the low rate, about half of the loci were unaffected by recombination, while at the medium or high rates, every locus had at least one recombination event.

Compared with recombination, other factors such as the number of sequences (S), the mutation rate (θ) and the number of loci (L) had greater impact on parameter estimation (Table 4). The number of loci (L) is the sample size in the model so that quadrupling the number of loci (L) may be expected to reduce the CI width by a half (e.g. White, 1982; O’Hagan & Forster, 2004, pp. 72–3). This expectation held for parameters that were well estimated, such as the species divergence times (Table S2).

## 4 DISCUSSION

### 4.1 Recombination hot spots and the impact of recombination

Genome‐wide recombination rate is known to vary over an order of magnitude among different eukaryotes (Stapley et al., 2017). Within the same species, recombination rate is known to vary between the sexes, and across the genome, with most crossovers occurring at the so‐called recombination hot spots (Jeffreys et al., 2001; Kauppi et al., 2004). Recombination hot spots were first documented in sperm‐typing experiments in humans and mice (Cullen et al., 1997, 2002) and confirmed in linkage‐disequilibrium (LD) analysis of single nucleotide polymorphism (SNP) markers or population genetic analysis of genomic sequences (Jeffreys et al., 2001; Myers et al., 2005). Technological advancements in the 2000s including efficient resequencing methods (which led to increased SNP marker density) and single‐molecule methods (which allow recombinant DNA molecules to be recovered directly from sperm DNA) have brought breakthroughs in studies of recombination hot spots. They have now been studied in a variety of species including fruit flies (Chan et al., 2012), cricket (Blankers et al., 2018), birds (Kawakami et al., 2017) and mammals (Jeffreys et al., 2001; see, for reviews, Kauppi et al., 2004; Arnheim et al., 2007; Penalba & Wolf, 2020). In humans, recombination hot spots are narrow regions of 1 to 2 kb, spaced on average every 50 to 100 kb, with highly variable levels of activity (Baudat et al., 2010; Myers et al., 2005).

In our simulation, we used the program ms, which assumes a constant recombination rate and does not allow the presence of recombination hot spots. Programs such as mshot (Hellenthal & Stephens, 2007) can simulate recombination hot spots. Based on estimates from the human being, a plausible simulation scenario may be to generate a mixture of loci, with, say, ~98% of them having the background recombination rate of ρ=0.05, and ~2% of hot spots with elevated rates of ρ=20 (Stapley et al., 2017; Wang & Rannala, 2009), with the average rate of ~0.45. It appears obvious that under such a scenario, the number of recombination events and the impact of recombination on the MSC‐based analyses will be less than that found in our simulation at ρ=0.5, and much less than in our simulation at ρ=5. Thus, we have not conducted simulation under a model of recombination hot spots.

Our simulation addresses the question whether it matters if recombination is ignored in the analysis when it is known to exist. The answer to this question obviously depends on the nature of the analysis and on the recombination rate. Here, we examined three major inference problems under the multispecies coalescent model using data of multilocus genomic sequence alignments: species tree estimation, species delimitation through Bayesian model selection and estimation of population parameters such as population sizes, species divergence times and cross‐species introgression probabilities. We found that the Bayesian methods for species tree estimation and species delimitation are robust to recombination even when the recombination rate is 10 times higher than the average human rate. We note that both those inference problems involve Bayesian model selection. For example, in the case of species tree estimation, the true model is the MSC model with recombination. We may then expect the true species tree with no recombination to be a less wrong model than any wrong species tree with no recombination, judged by the Kullback–Leibler divergence. Then, when the amount of data (the number of loci) approaches infinity, the less wrong model represented by the true species tree will dominate, with its posterior probability approaching 1 (Yang & Zhu, 2018). Bayesian estimation of species tree topology is then statistically ‘consistent’, in that the MAP model converges to the true species tree despite the model misspecification.

Such consistency or convergence does not apply to the problem of parameter estimation. When there is no recombination, the MSC model is correct, and the Bayesian point estimates (the posterior means) of all parameters will converge to the true values, and the CI width will converge to 0. However, when recombination is present and ignored, the model is misspecified. Then, the Bayesian estimates will converge to the best‐fitting parameters $\theta_{*}$, which differ from the true parameter values $\theta_{0}$, the CI width will converge to 0, but the CI coverage will become 0%. The difference, $\theta_{*}‐\theta_{0}$, measures the ‘bias’ in parameter estimation or the robustness of the analysis to model misspecification. As the information content and the variances of parameter estimates are expected to be nearly the same at different recombination rates when the rates are low, our results suggest that recombination at the low rates (10% of the human rate) had virtually no impact and produced nearly identical results as in the case of no recombination (Table S1). However, recombination at rates 10× higher than the average human rate produced biases in some parameters, with small biases (within 10% of true values) in population sizes for modern species and in species divergence times, but with much larger positive biases (20–40% of true values) in introgression times and in population sizes for species receiving migrants. The introgression probability was affected little even with such excessive recombination events. In summary, the bias in parameter estimation caused by ignoring recombination depends on the recombination rate and the parameters being estimated.

In the extreme case of an infinite recombination rate (ρ=∞), the different sites at the same locus will have independent genealogical histories. As a result, some parameters in the MSC and MSci models will become unidentifiable (Zhu & Yang, 2021). In the multilocus data sets, differences among sites of the same sequence reflect the random variations of the mutation process, while differences between loci reflect the stochastic fluctuation of the coalescent process. Thus, genealogical variations among loci provide important information about the parameters in the MSC model such as ancestral population sizes and species divergence times (Lohse & Frantz, 2014; Shi & Yang, 2018; Yang, 1997, 2002; Zhu & Yang, 2021). If the recombination rate between any pair of sites at the same locus is infinite, all sites in the data will have independent histories, and the two sources of variation will be confounded. As a result, the information from the coalescent variation among loci is lost, and some parameters in the model will become unidentifiable. The species tree topology remains identifiable by data of independent sites, but there is a dramatic loss of information (Zhu & Yang, 2021, Figure 3c). Nevertheless, the assumption of an infinite recombination rate between any sites in the sequence is implausible, and the performance of a method under such a model is not representative of its performance in real genomic data sets.

Overall, our simulation suggests that the impact of recombination on species tree estimation, species delimitation, and estimation of population parameters including species divergence times and cross‐species introgression probabilities, is relatively minor at realistic recombination rates. Species tree estimation is particularly robust to even excessive amounts of recombination, with over 50 recombination events in the genealogical history of one locus (500 bps) (Table 3, ρ=5). The results are consistent with the small‐scale simulation of Lanier and Knowles (2012). To understand this lack of effects, we examined the gene trees generated by ms for each locus in some of the simulated replicates. As discussed by Hein et al. (2005, pp. 148–150), a recombination event may (i) have no effect, (ii) change the branch lengths (coalescent times) or (iii) change both the tree topology and branch lengths. No theory is available for calculating the probabilities for those cases under the MSC model. Our examination of the simulated gene trees suggests that most recombination events caused no or little differences between the gene trees for the neighbouring segments of the same locus. For example, the first locus in the first replicate in the A01 simulation under tree B (Figure 1a) with S=2 sequences per species at the medium recombination rate (ρ=0.5) had 4 recombination events, breaking the 500‐bp locus into 5 segments of lengths 200, 59, 228, 11 and 2 sites, but the five gene trees had the same topology and branch lengths so that the recombination events were ‘invisible'. At the high rate (ρ=5) and with S=2, the first locus in the first replicate in the same setting had 72 recombination events, but the 73 gene trees had the same topology, sometimes with small differences in local branch lengths between neighbouring trees. At the high rate (ρ=5) and with S=8 sequences per species, the first locus in the first replicate had 73 recombination events, and the 74 gene trees had 20 distinct topologies, with all differences to concern the relationships among sequences from the same species. Thus, the recombination events in those simulations caused either no change to the gene tree, or the changes were minor and did not affect the relative support for the alternative species trees.

Nevertheless, our simulation has limited scope and our results should not be overgeneralized. For example, we used a fixed sequence length of 500 bps and three recombination rates that are within two orders of magnitude of the average human rate. If the recombination rate in the species group under study is much higher than the rates used here or if the sequence at each locus is much longer than 500 bps, the conclusions based on our simulation may not apply.

### 4.2 Information content and strategies for analysing genomic data sets

We note that our simulation does not address two related questions. The first is estimation of recombination rates (and identification of recombination hot spots). Recombination is an important biological process, and reliable estimation of recombination rates is critical for identification of disease‐causing mutations and detection of variants involved in selective sweeps (Clark, 2003; Penalba & Wolf, 2020). Second, even in the context of using mutations in the genome as neutral markers to infer the demographic history of species divergences, the MSC‐based methods are just one strategy for analysing the genomic data. By sampling short genomic segments that are far apart, recombination is ignored in the model, but the analysis does not utilize information in linkage disequilibrium (LD) between neighbouring segments of the genome, which may be informative about certain population genetic processes such as admixture. Alternately, a number of population genetic methods deal with recombination in the model explicitly and can be applied to large chromosomal regions. Examples include the sequential Markov coalescent approaches to inferring human population size and separation histories from multiple genomes (Li & Durbin, 2011; Schiffels & Durbin, 2014; Sheehan et al., 2013), the simulation‐based method of Wall (2003) for estimating species split times and ancestral population sizes under the MSC, the hidden Markov model (HMM) approach to estimating species divergence times and population sizes of Mailund et al. (2012). Most methods in this class use summary statistics such as the first‐coalescent time (Schiffels & Durbin, 2014) or the introgression haplotype tracks (Harris & Nielsen, 2013; Setter et al., 2020), or otherwise apply approximations to the ancestral recombination graphs (ARGs) (Griffiths & Marjoram, 1996; McVean & Cardin, 2005) because full‐likelihood implementations of the ARG are too costly (Wang and Rannala (2009).

The relative performance of the two strategies for analysing population genomic data is not well understood and appears to depend on the timescale. At very shallow timescales as in the analysis of different populations of the same species, there may be too few mutations in short genomic segments so that methods that leverage the information in LD may be advantageous. For data from different species, the phylogenomic methods based on the MSC that explicitly use information in the gene genealogies may be more powerful. Simulation may be useful to understand the relative power of the different classes of methods.

## CONFLICT OF INTEREST

The authors declare no conflict of interest.

## AUTHOR CONTRIBUTIONS

Z.Y. conceived the work, T.Z conducted the research, and Z.Y. wrote the paper. All authors read and approved the paper.

## BENEFITS GENERATED

Benefits from this research accrue from the sharing of our data and results on public databases as described above.

## Supporting information

Supplementary Material

## Data Availability

No new data were generated in this study. Supplementary information containing Figure S1, Tables S1 and S2, and supplementary text including MC simulation scripts and bpp control files for the three sets of simulations are available on Dryad (https://doi.org/10.5061/dryad.8w9ghx3p2).
