# Supplementary material for: A simulation study to examine the impact of recombination on phylogenomic inferences under the multispecies coalescent model
Source: Mol Ecol. 2022 Apr 4;31(10):2814–29. doi: 10.1111/mec.16433 (PMC9321900; doi:10.1111/mec.16433)
Supplement: Supplementary file 1 — Supplementary Material [file MEC-31-2814-s001.pdf]

MOLECULAR ECOLOGY

Supplemental Information for
A simulation study to examine the impact of recombination on inference using genomic sequence data under the multispecies coalescent model

Tianqi Zhu, Tomáš Flouri, and Ziheng Yang

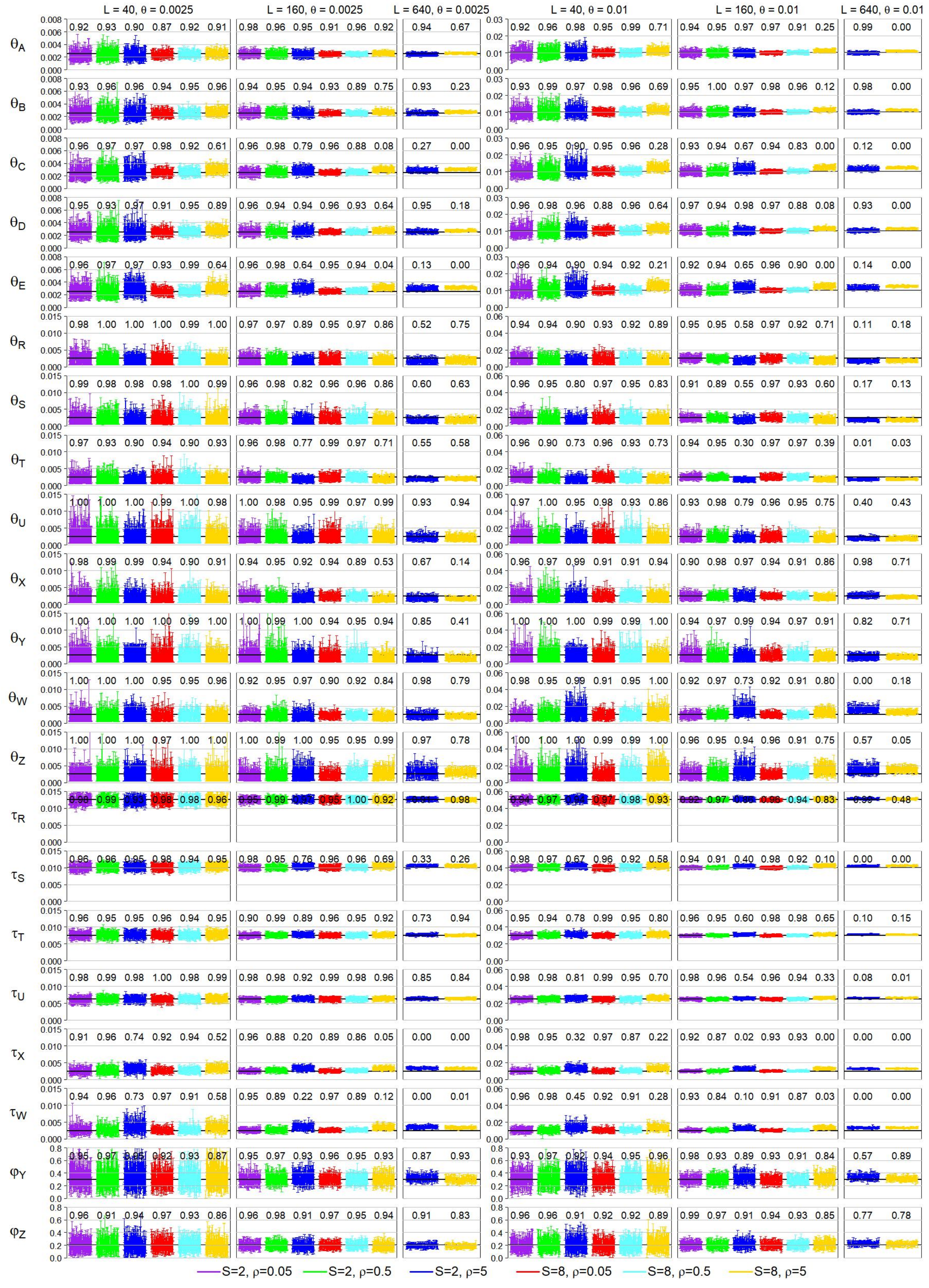

Figure S1. The posterior 95% CIs and CI coverage for parameters under the MSci model for species tree U of figure 3. Simulation for $L = 640$ loci was done for the high recombination rate ($\rho = 5$) only. See legend to figure 6.

Table S1. Averages parameter estimates over replicates divided by the true values in the A00 analysis under the MSci model

	ρ	θ_A	θ_B	θ_C	θ_D	θ_E	θ_R	θ_S	θ_T	θ_U	θ_X	θ_Y	θ_Z	θ_W	τ_R	τ_S	τ_T	τ_U	$\tau_X = \tau_Y$	$\tau_Z = \tau_W$	φ_Y	φ_W
**Tree B**																						
$\boldsymbol{\theta = 0.0025}$																						
$S = 2, L = 40$	0.05	0.98	1.03	1.01	1.03	1.01	0.9	1.03	0.91	1.24	0.97	0.99	0.94	0.99	1.01	1.01	1.01	0.99	1.06	1.06	1.04	1.02
	0.5	1.01	1.01	1.02	1.03	1.00	0.85	0.93	0.95	1.25	0.92	0.98	0.97	0.99	1.01	1.02	1.02	0.99	1.11	1.11	1.03	1.03
	5	0.99	1.04	1.20	1.06	1.18	0.77	0.84	0.90	1.3	0.92	0.97	0.87	0.94	1.03	1.03	1.03	0.94	1.43	1.4	1.05	0.98
$S = 2, L = 160$	0.05	0.99	1.01	1.02	1.02	1.03	0.95	1.03	0.96	1.2	0.94	0.97	0.91	0.96	1.00	1.00	1.01	1.00	1.04	1.05	1.00	1.00
	0.5	0.99	1.01	1.06	1.03	1.04	0.86	0.98	0.91	1.42	0.96	0.93	0.89	0.88	1.02	1.01	1.02	0.98	1.06	1.08	1.00	0.99
	5	1.01	1.03	1.23	1.03	1.21	0.69	0.8	0.79	1.23	0.97	0.94	0.86	0.85	1.04	1.03	1.05	0.96	1.42	1.34	1.13	0.87
$S = 8, L = 40$	0.05	0.99	1.01	1.02	1.00	1.01	0.90	0.94	1.01	1.22	0.93	0.96	0.94	0.91	1.01	1.01	1.01	0.99	1.06	1.08	1.02	1.04
	0.5	0.99	1.03	1.05	1.01	1.06	0.86	0.96	0.99	1.22	0.95	0.99	0.90	0.97	1.01	1.01	1.01	0.98	1.07	1.10	1.00	1.02
	5	1.03	1.11	1.27	1.08	1.22	0.77	0.90	0.85	1.29	0.83	0.92	0.83	0.90	1.03	1.01	1.04	0.95	1.4	1.33	1.03	0.90
$S = 8, L = 160$	0.05	0.99	0.99	1.00	1.00	1.02	0.93	1.00	0.96	1.19	1.01	0.94	0.92	0.94	1.01	1.00	1.01	0.99	1.02	1.05	0.97	1.00
	0.5	1.01	1.02	1.04	1.01	1.03	0.87	0.99	0.92	1.25	0.95	1.03	0.96	0.97	1.01	1.00	1.01	0.99	1.05	1.04	1.01	1.00
	5	1.05	1.10	1.27	1.09	1.23	0.69	0.77	0.77	1.68	0.85	0.9	0.81	0.88	1.04	1.03	1.05	0.93	1.41	1.26	1.04	0.86
$\boldsymbol{\theta = 0.01}$																						
$S = 2, L = 40$	0.05	0.97	1.03	1.01	0.99	0.98	0.95	0.95	0.96	1.12	0.97	0.97	0.95	0.96	1.00	1.01	1.00	1.00	1.03	1.08	1.05	0.97
	0.5	0.98	1.01	1.06	1.03	1.04	0.89	0.95	0.91	1.18	0.96	0.98	0.89	0.95	1.01	1.01	1.02	0.99	1.08	1.08	1.00	1.00
	5	0.98	1.01	1.19	1.03	1.18	0.70	0.76	0.71	1.45	1.10	1.02	1.11	0.94	1.03	1.03	1.06	0.95	1.38	1.33	1.09	1.01
$S = 2, L = 160$	0.05	0.99	1.01	1.01	1.00	1.01	0.98	1.02	0.99	1.07	0.95	1.03	0.98	1.02	1.00	1.00	1.00	1.00	1.02	1.02	0.99	0.97
	0.5	1.00	1.01	1.02	1.00	1.03	0.92	0.97	0.95	1.17	0.99	1.02	0.99	0.98	1.01	1.00	1.01	0.99	1.04	1.05	0.99	1.00
	5	0.99	1.03	1.21	1.03	1.18	0.68	0.75	0.68	1.36	1.25	1.11	1.26	0.95	1.04	1.03	1.06	0.96	1.29	1.33	1.10	1.02
$S = 8, L = 40$	0.05	1.00	1.00	1.02	1.01	1.01	0.96	0.98	0.97	1.16	0.95	0.99	0.93	1.02	1.00	1.01	1.01	0.99	1.02	1.03	0.99	1.00
	0.5	1.00	1.01	1.04	1.02	1.04	0.91	0.97	0.89	1.25	0.96	1.09	0.99	0.99	1.01	1.01	1.02	0.99	1.06	1.08	1.02	1.03
	5	1.11	1.12	1.27	1.13	1.25	0.69	0.80	0.76	1.57	1.05	1.09	1.01	1.05	1.04	1.02	1.07	0.94	1.37	1.34	1.09	0.98
$S = 8, L = 160$	0.05	1.00	1.00	1.00	1.01	1.01	0.98	0.98	0.98	1.08	0.99	0.99	0.96	0.97	1.00	1.01	1.00	1.00	1.02	1.01	1.02	1.01
	0.5	1.02	1.01	1.03	1.01	1.03	0.93	0.99	0.93	1.10	1.02	1.04	1.01	1.03	1.01	1.00	1.01	1.00	1.04	1.04	1.01	1.01
	5	1.11	1.14	1.27	1.14	1.25	0.67	0.78	0.69	1.82	1.11	1.14	1.07	1.08	1.05	1.02	1.07	0.93	1.32	1.26	1.06	0.99
**Tree U**																						
$\boldsymbol{\theta = 0.0025}$																						
$S = 2, L = 40$	0.05	0.93	0.99	1.01	1.02	1.02	1.04	0.94	0.94	1.18	0.95	1.02	0.92	0.94	0.99	1.01	1.01	0.99	1.04	1.10	1.10	1.00
	0.5	0.98	1.01	1.03	1.00	1.03	0.88	0.91	0.92	1.09	0.98	0.96	0.93	0.96	1.00	1.02	1.03	1.00	1.10	1.08	1.06	1.01
	5	0.95	1.01	1.11	1.01	1.18	0.83	0.82	0.78	1.01	0.84	0.91	0.89	0.99	0.99	1.02	1.03	1.00	1.31	1.5	1.15	0.98
$S = 2, L = 160$	0.05	1.00	1.01	1.00	1.02	1.00	0.94	1.00	0.99	1.04	0.96	0.99	0.9	0.92	1.01	1.00	1.00	0.99	1.02	1.05	1.00	1.00
	0.5	0.99	1.02	1.03	0.99	1.03	0.89	0.95	0.94	1.08	0.91	1.03	0.99	1.03	1.01	1.01	1.01	1.00	1.09	1.06	1.03	1.01
	5	0.98	1.02	1.14	1.05	1.21	0.73	0.77	0.75	0.90	0.80	0.82	0.88	1.11	1.01	1.04	1.04	1.03	1.36	1.46	1.10	0.96
$S = 8, L = 40$	0.05	0.97	1.00	1.01	1.00	1.01	0.95	0.92	0.96	1.12	0.91	1.00	0.94	0.99	0.99	1.01	1.01	0.99	1.06	1.07	1.04	1.02
	0.5	0.99	1.00	1.05	1.03	1.03	0.93	0.88	0.9	1.09	0.93	0.97	0.92	0.97	1.00	1.02	1.01	1.00	1.09	1.12	1.04	1.02
	5	1.02	1.09	1.22	1.10	1.23	0.79	0.87	0.8	0.97	0.79	0.86	0.81	1.03	1.00	1.04	1.04	1.03	1.37	1.42	1.17	0.93
$S = 8, L = 160$	0.05	0.99	1.00	1.01	1.01	1.01	0.89	0.96	0.98	1.06	0.95	0.92	0.93	0.91	1.00	1.01	1.00	1.00	1.02	1.03	1.00	0.99
	0.5	1.00	1.02	1.04	1.02	1.04	0.84	0.92	0.98	0.98	0.93	0.93	0.91	1.03	1.01	1.02	1.01	1.01	1.06	1.07	1.00	1.02
	5	1.05	1.09	1.21	1.10	1.25	0.74	0.76	0.79	0.92	0.67	0.74	0.80	1.11	1.01	1.05	1.02	1.03	1.35	1.4	1.03	0.94
$\boldsymbol{\theta = 0.01}$																						
$S = 2, L = 40$	0.05	0.97	0.98	1.03	1.01	1.05	0.98	0.95	1.04	1.02	0.99	0.96	0.96	1.03	1.00	1.00	1.00	1.00	1.04	1.06	1.01	1.00
	0.5	1.00	1.04	1.01	0.99	1.01	0.89	0.92	0.92	0.98	1.03	0.98	0.97	1.03	1.01	1.02	1.02	1.01	1.08	1.06	1.03	1.05
	5	0.99	1.05	1.18	1.04	1.18	0.76	0.74	0.74	0.91	0.94	0.91	1.29	1.17	1.01	1.05	1.05	1.05	1.35	1.42	1.12	1.07
$S = 2, L = 160$	0.05	0.98	0.99	1.01	1.03	1.00	1.02	0.96	1.02	0.98	0.95	0.96	0.96	0.97	1.00	1.00	1.00	1.00	1.02	1.04	0.99	1.02
	0.5	1.00	1.02	1.03	0.99	1.01	0.94	0.94	0.97	0.99	0.97	0.96	1.03	1.05	1.00	1.01	1.00	1.01	1.04	1.06	1.00	1.01
	5	0.98	1.01	1.16	1.02	1.17	0.72	0.74	0.71	0.8	1.00	0.84	1.58	1.34	1.02	1.05	1.04	1.05	1.32	1.32	1.12	1.09
$S = 8, L = 40$	0.05	1.01	1.01	1.01	1.02	1.00	0.95	0.96	0.97	1.02	0.96	0.95	0.93	0.95	1.00	1.00	1.00	1.00	1.03	1.06	1.01	0.97
	0.5	1.01	1.03	1.03	1.01	1.03	0.89	0.95	0.91	1.01	0.95	1.04	0.99	1.04	1.00	1.01	1.01	1.00	1.07	1.08	1.04	0.99
	5	1.13	1.14	1.25	1.14	1.27	0.79	0.77	0.75	0.82	0.86	0.86	1.15	1.33	1.02	1.06	1.05	1.07	1.36	1.38	1.06	1.07
$S = 8, L = 160$	0.05	1.00	1.00	1.01	1.00	1.01	0.98	1.00	1.00	1.00	0.97	0.97	1.00	0.96	1.00	1.00	1.00	1.00	1.01	1.01	0.98	1.02
	0.5	1.01	1.02	1.03	1.02	1.03	0.93	0.94	1.00	0.95	0.98	0.98	0.99	1.14	1.00	1.01	1.00	1.01	1.03	1.05	0.99	1.02
	5	1.12	1.14	1.24	1.15	1.26	0.75	0.75	0.75	0.79	0.86	0.84	1.29	1.51	1.02	1.06	1.04	1.06	1.30	1.34	1.06	1.06

Note.— The value 1.12 means a positive bias of 12% (of the true value) while 0.75 means a negative bias of 25%.

Table S2. rRMSE of parameter estimates in the A00 analysis under the MSci model

	ρ	θ_A	θ_B	θ_C	θ_D	θ_E	θ_R	θ_S	θ_T	θ_U	θ_X	θ_Y	θ_Z	θ_W	τ_R	τ_S	τ_T	τ_U	$\tau_X = \tau_Y$	$\tau_Z = \tau_W$	φ_Y	φ_W
**Tree B**																						
$\theta = 0.0025$																						
$S = 2, L = 40$	0.05	0.20	0.23	0.22	0.23	0.22	0.26	0.32	0.27	0.37	0.31	0.27	0.28	0.36	0.04	0.06	0.06	0.06	0.21	0.28	0.22	0.30
	0.5	0.19	0.24	0.22	0.22	0.22	0.28	0.29	0.38	0.44	0.32	0.27	0.32	0.28	0.04	0.06	0.07	0.07	0.25	0.30	0.27	0.34
	5	0.16	0.23	0.34	0.26	0.32	0.30	0.25	0.35	0.53	0.28	0.15	0.21	0.16	0.04	0.06	0.08	0.09	0.51	0.56	0.29	0.30
$S = 2, L = 160$	0.05	0.11	0.11	0.12	0.13	0.11	0.22	0.26	0.24	0.42	0.24	0.40	0.24	0.35	0.02	0.03	0.04	0.04	0.12	0.14	0.12	0.16
	0.5	0.1	0.12	0.13	0.12	0.12	0.25	0.22	0.26	1.15	0.22	0.28	0.25	0.29	0.03	0.04	0.05	0.06	0.12	0.16	0.13	0.17
	5	0.09	0.11	0.26	0.11	0.25	0.35	0.28	0.30	0.58	0.23	0.24	0.27	0.27	0.04	0.05	0.06	0.06	0.44	0.39	0.19	0.25
$S = 8, L = 40$	0.05	0.1	0.11	0.11	0.11	0.12	0.25	0.31	0.36	0.34	0.29	0.36	0.27	0.28	0.04	0.06	0.07	0.05	0.17	0.23	0.25	0.35
	0.5	0.11	0.13	0.13	0.12	0.12	0.30	0.32	0.33	0.35	0.27	0.37	0.28	0.29	0.03	0.05	0.07	0.06	0.17	0.23	0.22	0.31
	5	0.12	0.17	0.31	0.15	0.27	0.29	0.33	0.28	0.6	0.25	0.19	0.27	0.25	0.04	0.06	0.06	0.09	0.45	0.43	0.29	0.30
$S = 8, L = 160$	0.05	0.05	0.06	0.06	0.06	0.07	0.20	0.26	0.26	0.49	0.21	0.29	0.20	0.29	0.02	0.04	0.04	0.03	0.08	0.11	0.11	0.16
	0.5	0.06	0.07	0.07	0.06	0.07	0.24	0.26	0.26	0.49	0.18	0.28	0.17	0.26	0.03	0.04	0.05	0.04	0.09	0.10	0.11	0.16
	5	0.07	0.12	0.28	0.11	0.25	0.34	0.31	0.29	1.27	0.23	0.24	0.28	0.25	0.04	0.04	0.06	0.10	0.42	0.31	0.15	0.21
$\theta = 0.01$																						
$S = 2, L = 40$	0.05	0.16	0.19	0.17	0.19	0.18	0.23	0.28	0.28	0.33	0.26	0.32	0.27	0.30	0.02	0.03	0.04	0.03	0.13	0.20	0.24	0.27
	0.5	0.15	0.18	0.21	0.19	0.18	0.27	0.28	0.26	0.47	0.29	0.31	0.29	0.29	0.03	0.04	0.04	0.04	0.15	0.18	0.24	0.31
	5	0.14	0.14	0.30	0.18	0.28	0.33	0.32	0.33	0.98	0.29	0.26	0.34	0.26	0.04	0.05	0.07	0.09	0.43	0.41	0.27	0.33
$S = 2, L = 160$	0.05	0.08	0.09	0.11	0.09	0.1	0.11	0.19	0.17	0.36	0.18	0.35	0.20	0.29	0.01	0.02	0.03	0.02	0.07	0.07	0.12	0.15
	0.5	0.09	0.1	0.11	0.09	0.11	0.14	0.16	0.20	0.49	0.16	0.38	0.21	0.27	0.01	0.02	0.03	0.04	0.07	0.10	0.12	0.15
	5	0.08	0.09	0.24	0.09	0.21	0.34	0.30	0.34	0.80	0.31	0.31	0.39	0.26	0.04	0.04	0.07	0.06	0.30	0.35	0.17	0.19
$S = 8, L = 40$	0.05	0.08	0.07	0.08	0.08	0.09	0.21	0.34	0.25	0.44	0.22	0.35	0.26	0.29	0.02	0.03	0.04	0.04	0.09	0.13	0.20	0.26
	0.5	0.08	0.1	0.10	0.09	0.09	0.24	0.27	0.25	0.62	0.24	0.36	0.25	0.28	0.03	0.04	0.04	0.04	0.12	0.15	0.22	0.29
	5	0.14	0.15	0.30	0.16	0.27	0.35	0.31	0.37	1.05	0.22	0.31	0.26	0.27	0.05	0.05	0.08	0.1	0.41	0.40	0.27	0.32
$S = 8, L = 160$	0.05	0.03	0.04	0.04	0.04	0.04	0.12	0.17	0.17	0.31	0.14	0.23	0.14	0.18	0.01	0.02	0.02	0.02	0.04	0.05	0.11	0.13
	0.5	0.04	0.04	0.05	0.04	0.06	0.13	0.19	0.16	0.39	0.12	0.22	0.16	0.19	0.01	0.02	0.03	0.03	0.06	0.08	0.11	0.13
	5	0.11	0.15	0.28	0.15	0.25	0.35	0.27	0.33	1.29	0.18	0.27	0.22	0.21	0.05	0.03	0.08	0.1	0.32	0.28	0.15	0.18
**Tree U**																						
$\theta = 0.0025$																						
$S = 2, L = 40$	0.05	0.20	0.21	0.21	0.21	0.22	0.35	0.27	0.27	0.42	0.29	0.27	0.32	0.27	0.05	0.05	0.06	0.06	0.21	0.35	0.32	0.29
	0.5	0.19	0.21	0.22	0.25	0.22	0.26	0.28	0.27	0.33	0.36	0.23	0.28	0.27	0.05	0.05	0.08	0.07	0.22	0.29	0.30	0.38
	5	0.19	0.20	0.24	0.21	0.28	0.26	0.26	0.29	0.26	0.27	0.17	0.22	0.23	0.05	0.05	0.07	0.06	0.38	0.64	0.35	0.36
$S = 2, L = 160$	0.05	0.1	0.12	0.11	0.12	0.12	0.27	0.25	0.22	0.27	0.28	0.43	0.26	0.28	0.03	0.03	0.04	0.05	0.11	0.13	0.17	0.15
	0.5	0.1	0.12	0.11	0.13	0.12	0.25	0.27	0.17	0.36	0.27	0.41	0.29	0.37	0.03	0.04	0.04	0.05	0.15	0.17	0.16	0.16
	5	0.09	0.11	0.18	0.14	0.25	0.31	0.31	0.28	0.27	0.28	0.24	0.24	0.34	0.03	0.05	0.05	0.05	0.38	0.50	0.22	0.21
$S = 8, L = 40$	0.05	0.12	0.12	0.11	0.13	0.12	0.27	0.27	0.30	0.36	0.33	0.35	0.30	0.38	0.05	0.04	0.07	0.07	0.18	0.19	0.29	0.27
	0.5	0.11	0.12	0.13	0.12	0.11	0.27	0.29	0.30	0.36	0.33	0.31	0.29	0.27	0.05	0.05	0.06	0.06	0.18	0.26	0.30	0.29
	5	0.12	0.14	0.25	0.17	0.29	0.27	0.29	0.27	0.24	0.30	0.23	0.26	0.41	0.05	0.06	0.07	0.06	0.42	0.52	0.42	0.36
$S = 8, L = 160$	0.05	0.06	0.06	0.06	0.06	0.06	0.25	0.26	0.17	0.29	0.18	0.30	0.20	0.27	0.03	0.03	0.03	0.04	0.09	0.09	0.13	0.13
	0.5	0.05	0.07	0.07	0.07	0.07	0.26	0.27	0.17	0.27	0.20	0.32	0.20	0.31	0.03	0.03	0.04	0.04	0.10	0.12	0.14	0.16
	5	0.07	0.11	0.22	0.12	0.26	0.31	0.29	0.26	0.24	0.36	0.30	0.27	0.33	0.03	0.06	0.05	0.04	0.36	0.42	0.19	0.18
$\theta = 0.01$																						
$S = 2, L = 40$	0.05	0.16	0.19	0.19	0.18	0.22	0.27	0.26	0.26	0.31	0.32	0.29	0.26	0.34	0.04	0.03	0.04	0.04	0.12	0.15	0.25	0.27
	0.5	0.14	0.20	0.20	0.17	0.19	0.27	0.29	0.26	0.28	0.38	0.27	0.28	0.36	0.03	0.03	0.04	0.05	0.15	0.17	0.25	0.29
	5	0.15	0.17	0.28	0.17	0.28	0.29	0.32	0.31	0.29	0.28	0.16	0.49	0.44	0.04	0.06	0.07	0.07	0.39	0.49	0.31	0.38
$S = 2, L = 160$	0.05	0.09	0.09	0.1	0.09	0.1	0.18	0.17	0.15	0.25	0.22	0.31	0.21	0.26	0.02	0.02	0.02	0.03	0.07	0.09	0.12	0.14
	0.5	0.08	0.08	0.1	0.09	0.1	0.16	0.18	0.13	0.23	0.19	0.29	0.20	0.30	0.02	0.02	0.02	0.03	0.09	0.11	0.13	0.15
	5	0.07	0.08	0.19	0.08	0.20	0.32	0.31	0.30	0.28	0.19	0.29	0.67	0.54	0.03	0.05	0.04	0.05	0.33	0.34	0.19	0.22
$S = 8, L = 40$	0.05	0.08	0.08	0.08	0.1	0.09	0.27	0.25	0.20	0.31	0.27	0.29	0.25	0.27	0.03	0.03	0.03	0.04	0.11	0.13	0.23	0.28
	0.5	0.07	0.08	0.09	0.08	0.09	0.27	0.27	0.21	0.37	0.24	0.34	0.26	0.36	0.03	0.04	0.04	0.05	0.13	0.16	0.23	0.28
	5	0.16	0.16	0.27	0.17	0.30	0.29	0.30	0.30	0.29	0.24	0.26	0.36	0.53	0.04	0.07	0.06	0.08	0.40	0.43	0.28	0.37
$S = 8, L = 160$	0.05	0.04	0.04	0.05	0.04	0.04	0.17	0.16	0.13	0.22	0.14	0.24	0.16	0.21	0.02	0.02	0.02	0.03	0.05	0.07	0.11	0.15
	0.5	0.05	0.04	0.06	0.05	0.05	0.19	0.16	0.13	0.23	0.15	0.21	0.16	0.30	0.02	0.02	0.02	0.03	0.05	0.08	0.11	0.15
	5	0.12	0.15	0.25	0.15	0.27	0.28	0.30	0.27	0.26	0.19	0.22	0.35	0.58	0.03	0.06	0.04	0.31	0.07	0.35	0.17	0.20

Supplementary text. MS Simulation Scripts and BPP Control Files

A01 Species tree estimation

MS simulation scripts

```
Tree B
ms 10 L -r rho 500 -T -I 5 2 2 2 2 2 -ej 4.7 2 1 -ej 4.8 3 1 -ej 4.8 4 5 -ej 5 5 1|grep -v
// >treefile
seq-gen -mHKY -l 500 -s theta -p 200 <treefile >seqfile.txt


Tree U
ms 10 L -r rho 500 -T -I 5 2 2 2 2 2 -ej 4.4 2 1 -ej 4.6 3 1 -ej 4.8 4 1 -ej 5 5 1|grep -v
// >treefile
seq-gen -mHKY -l 500 -s theta -p 200 <treefile >seqfile.txt
```

bpp control file

```
seed = -1
seqfile = seqfile.txt
Imapfile = ../../Imap.txt
outfile = out.txt
mcmcfile = mcmc.txt

speciesdelimitation = 0
speciestree = 1
speciesmodelprior = 1
species&tree = 5   A  B  C  D  E
                    2  2  2  2  2
*                   8  8  8  8  8
 (((A, B), C), ( D, E)) ;  #treeB
*((((A, B), C), D), E);  #treeU

usedata = 1
nloci = 160  #40
cleandata = 0
thetaprior = 3 0.02   #  3 0.005  # e没了
tauprior = 3 0.1     #  3 0.025
*phiprior = 0.3  0.7
finetune = 1: 21.06 .0002 .0003 .00001 .2 .01 .01 .01
print = 1 0 0 0
burnin = 32000
sampfreq = 5
nsample = 100000
```

A11 Species delimitation

MS simulation scripts

```
Tree D (Deep)
ms 10 L -r rho 500 -T -I 5 2 2 2 2 2 -ej 1E-50 2 1 -ej 1E-50 4 5 -ej 4.8 3 1 -ej 5 5 1|grep
-v // >treefile
seq-gen -mHKY -l 500 -s theta -p 200 <treefile >seqfile.txt


Tree S (Shallow)
ms 10 L -r rho 500 -T -I 5 2 2 2 2 2 -ej 1E-50 2 1 -ej 1E-50 4 5 -ej 0.5 3 1 -ej 1 5 1|grep
-v // >treefile
seq-gen -mHKY -l 500 -s theta -p 200 <treefile >seqfile.txt
```

bpp control file

```
seed = -1
seqfile = seqfile.txt
Imapfile = ../../Imap.txt
outfile = out.txt
mcmcfile = mcmc.txt

speciesdelimitation = 1 0 2
speciestree = 1
speciesmodelprior = 1
species&tree = 5   A  B  C  D  E
                    2  2  2  2  2
*                   8  8  8  8  8
                 (((A, B), C), ( D, E)) ;

usedata = 1
nloci = 160  #40
cleandata = 0
thetaprior = 3 0.02   #  3 0.005
tauprior = 3 0.1
#Deep Tree: theta = 0.01 ; tauprior = 3 0.1; theta = 0.025, tauprior = 3 0.025
#Shallow Tree: theta = 0.01; tauprior = 3 0.02; theta = 0.025, tauprior = 3 0.005

finetune = 1: 21.06 .0002 .0003 .00001 .2 .01 .01 .01
print = 1 0 0 0
burnin = 32000
sampfreq = 5
nsample = 100000
```

A00 Estimation of population parameters under the MSci model

MS simulation scripts

```
Tree B
ms 10 L -r rho 500 -T -I 5 2 2 2 2 2 -es 1 3 0.7 -es 1 5 0.8 -ej 1.0001 6 2 -ej 1.0001 7 4
-en 1.0002 2 1 -en 1.0002 3 1 -en 1.0002 4 1 -en 1.0002 5 1 -ej 3 2 1 -ej 4 3 1 -ej 4.5 4 5
-ej 5 5 1|grep -v // >treefile
seq-gen -mHKY -l 500 -s theta -p 200 <treefile >seqfile.txt
# ms 40 L -r rho 500 -T -I 5 8 8 8 8 8 for S = 8

Tree U
ms 10 L -r rho 500 -T -I 5 2 2 2 2 2 -es 1 3 0.7 -es 1 5 0.8 -ej 1.0001 6 2 -ej 1.0001 7 4
-en 1.0002 2 1 -en 1.0002 3 1 -en 1.0002 4 1 -en 1.0002 5 1 -ej 2.5 2 1 -ej 3 3 1 -ej 4 4 1
-ej 5 5 1|grep -v // >treefile
seq-gen -mHKY -l 500 -s theta -p 200 <treefile >seqfile.txt
# ms 40 L -r rho 500 -T -I 5 8 8 8 8 8 for S = 8
```

bpp control file

```
seed = -1
seqfile = seqfile.txt
Imapfile = Imap.txt
outfile = out.txt
mcmcfile = mcmc.txt

speciesdelimitation = 0
speciestree = 0
speciesmodelprior = 1
species&tree = 5   A  B  C  D  E
                   2  2  2  2  2
*                  8  8  8  8  8
(((A,(B,(C)Y[&tau-parent=no])X)T,Y[&tau-parent=yes])S, (( D, (E)W[&tau-parent=no])Z,W[&tau-
parent=yes])U)R ;  #treeB
*((((A, (B, (C) Y[&tau-parent=no]) X) U, Y[&tau-parent=yes]) T, (D, (E) W[&tau-parent=no])
Z) S, W[&tau-parent=yes]) R;  #treeU

usedata = 1
nloci = 160  #40
cleandata = 0
thetaprior = 3 0.02 e  #  3 0.005 e
tauprior = 3 0.1     #  3 0.025
phiprior = 0.3  0.7
finetune = 1: 21.06 .0002 .0003 .00001 .2 .01 .01 .01
print = 1 0 0 0
burnin = 32000
sampfreq = 5
nsample = 100000
```
